# Supplementary material for: Chromothripsis during telomere crisis is independent of NHEJ, and consistent with a replicative origin
Source: Genome Res. 2019 May;29(5):737–49. doi: 10.1101/gr.240705.118 (PMC6499312; doi:10.1101/gr.240705.118)
Supplement: Supplemental Material [file supp_gr.240705.118_Supplemental_file_1.zip › contigs/annotated_contigs/DB51/contig.2.DB51_length_556_mean_cov_6.11870503597.docx]

**DB51_length_556_mean_cov_6.11870503597**

```
TAACCAGAACAATTAGCTCCTATTATTACAACACATTAATATATCTTCATCTTCCAAAAGTTCTGCATAATTACATTTCTACAAAATTG
 >chr3:164342108-164342453 - E=5e-193
CCTATACAATTTGAACTTCATTCCTAAATTGATTTTTTTGGGGGAATGGGTGTTATTTTTTCTAAAGCAATGTTACATATTTTTTGTTT

TATCTTTAATTTTATTGCCTTCTGTTCAGAGATTATTGGTGATAACTATGCAGATTATTATAAAGTTTCACAATTTAATATAATTTTGA

AAGGATATTGCATTGACATTATGAATTTTTAGCATTATCTTAAATATAATAATGAACATATATGGAAGAATGACATAT|ACTTTTACAT

GTATATGTATAAG|TAAATTGGCAGTTATCAGCATGGCTAATTCATCAGATGTTACTTGTATCATAGTGTGTAGAATGTCTTTTTTTTA
              >chr3:164340477-164340665 - E=2e-101
CACCTTCTGATTTTCATTAGTTTATCTATGTCTCTATAGATCATAATTTATTTGAATATATTTTTAAATACTTCTCATACATTCTACAT

ACATATTTTGCTATTCTTAAAGTT
```
